# Truncated tau deregulates synaptic markers in rat model for human tauopathy

**DOI:** 10.3389/fncel.2015.00024

**Published:** 2015-02-23

**Authors:** Santosh Jadhav, Stanislav Katina, Andrej Kovac, Zuzana Kazmerova, Michal Novak, Norbert Zilka

**Affiliations:** ^1^Institute of Neuroimmunology, Slovak Academy of SciencesBratislava, Slovak Republic; ^2^Axon Neuroscience GmbHBratislava, Slovak Republic; ^3^Institute of Mathematics and Statistics, Masaryk UniversityBrno, Czech Republic

**Keywords:** Alzheimer’s disease, truncated tau, phosphorylation, synaptic damage, tau mislocalization

## Abstract

Synaptic failure and neurofibrillary degeneration are two major neuropathological substrates of cognitive dysfunction in Alzheimer’s disease (AD). Only a few studies have demonstrated a direct relationship between these two AD hallmarks. To investigate tau mediated synaptic injury we used rat model of tauopathy that develops extensive neurofibrillary pathology in the cortex. Using fractionation of cortical synapses, we identified an increase in endogenous rat tau isoforms in presynaptic compartment, and their mis-sorting to the postsynaptic density (PSD). Truncated transgenic tau was distributed in both compartments exhibiting specific phospho-pattern that was characteristic for each synaptic compartment. In the presynaptic compartment, truncated tau was associated with impairment of dynamic stability of microtubules which could be responsible for reduction of synaptic vesicles. In the PSD, truncated tau lowered the levels of neurofilaments. Truncated tau also significantly decreased the synaptic levels of Aβ40 but not Aβ42. These data show that truncated tau differentially deregulates synaptic proteome in pre- and postsynaptic compartments. Importantly, we show that alteration of Aβ can arise downstream of truncated tau pathology.

## Introduction

Neurofibrillary tangles, amyloid plaques, neuronal loss and synaptic failure represent the major hallmarks of Alzheimer’s disease (AD; Wischik et al., 1988; Rinne et al., 1989; DeKosky and Scheff, 1990; Cras et al., 1991; West et al., 1994; Coleman and Yao, 2003). Synaptic deficits occur very early in AD and correlate well with the severity of dementia in AD patients (Davies et al., 1987; Masliah et al., 2001; Scheff et al., 2007). Structural and functional synaptic changes are observed in both early and late stages of AD (Sze et al., 2000; Masliah et al., 2001; Honer, 2003; Reddy et al., 2005; Counts et al., 2006). Moreover, these changes correlate well with the cognitive decline in AD (DeKosky and Scheff, 1990; Braak and Braak, 1991; Arriagada et al., 1992; Blennow et al., 1996; Callahan et al., 2002).

The synaptic damage is characterized by deregulation of synaptic proteins at the protein and mRNA levels (Coleman and Yao, 2003; Honer, 2003; Tao et al., 2003). For example, the level of synaptophysin—a presynaptic vesicle protein—decreased by 25% in the frontal cortex (Masliah et al., 2001) and by 35% in the superior temporal and inferior parietal cortex in AD (Counts et al., 2006). Decrease in synaptogamin by 52%, and synaptobrevin by 46% in hippocampus and 37% in the occipital cortex have also been demonstrated (Sze et al., 2000). Moreover, the vesicle protein Rab3a is decreased by 30% in hippocampus in AD brains (Sze et al., 2000). Several other proteins such as contactin I and II, SNAP 25, syntaxin are also deregulated in AD brains (Honer, 2003; Tannenberg et al., 2006). In addition, the surviving neurons in AD exhibit decreased levels of mRNA expressing synaptic proteins (Heffernan et al., 1998; Coleman and Yao, 2003). Loss of synaptic proteins, therefore, appears to dictate synaptic impairment in human AD.

Importantly, the pattern of NFT progression parallels synaptic damage and occurs in the same brain regions (Honer et al., 1992; Masliah et al., 1992; Wakabayashi et al., 1994; Ingelsson et al., 2004; Serrano-Pozo et al., 2011). Higher tangle count is associated with abatement in synaptic proteins in AD brains (Coleman et al., 1992; Callahan et al., 1994; Callahan and Coleman, 1995; Honer, 2003). Furthermore, synaptic failure is also reported in tauopathies such as frontal lobe degeneration and progressive supranuclear palsy (Brun et al., 1995; Bigio et al., 2001). Pathological tau then may lead directly to synaptic pathology in AD and related tauopathies. How the disease modified tau protein induces synaptic damage, however, remains unknown. Here we employed rat model of human tauopathy expressing human truncated tau that recapitulates AD neurofibrillary degeneration in the isocortex (Filipcik et al., 2012). Previously, we introduced truncated tau as a driving force behind neurofibrillary degeneration. Monoclonal antibody DC11 that recognized conformationally altered truncated tau protein (Vechterova et al., 2003) allowed us to identify several truncated tau species with toxic gain of function. Using *in vitro* tau oligomerization assay we selected the most toxic one that was used as a transgene for generation of rat models for human tauopathies (Zilka et al., 2006; Koson et al., 2008; Filipcik et al., 2012). The expression of the truncated tau in the brain of transgenic animals induced the complete tau cascade of neurofibrillary degeneration as found in humans. Truncated tau induced tau hyperphosphorylation, formation of Gallyas-positive intracellular and extracellular tangles also exhibiting Congo Red birefringence and thioflavin S reactivity, formation of sarkosyl-insoluble misfolded tau complexes containing both truncated and endogenous rat tau (Zilka et al., 2006; Filipcik et al., 2012). In this study, we demonstrate that truncated tau selectively alters synaptic tau, amyloid and cytoskeletal proteins in the pre- and postsynaptic compartments. Our findings suggest that truncated tau is a potent inducer of synaptic damage synapses independent of Aβ pathology.

## Materials and methods

### Transgenic rats

Heterozygous transgenic male rats expressing human N- and C-terminally truncated tau encompassing three repeats (aa 151–391; line SHR24; Filipcik et al., 2012) and age matched wild type rats were used in this study. Rats were housed in cages with adequate supply of water, and 12 h day/light cycle. Animals in the age of 14–16 months were sacrificed by cervical dislocation and brains were isolated and frozen. For each experiment, four animals per group were used; three animals were used for electron microscopy (EM). Left cortex was used for synaptosomal fractionation and right cortex was used for isolation of sarkosyl insoluble tau. All experiments were performed in accordance to the Slovak and European Community Guidelines, with the approval of the Ethical Committee of Institute of Neuroimmunology and the State Veterinary and Food Administration of the Slovak Republic.

### Antibodies

Primary antibodies used in this study and corresponding dilutions are provided in Table 1. Antibodies were diluted in fat free milk or BSA in 1 × Tris buffered saline with tween 20 or according to instructions provided by the manufacturer. Secondary antibodies were purchased from DAKO (DAKO, Glostrup, Denmark).

**Table 1:** **List of primary antibodies, clonality, and dilution used**.

Antibody (clone)	Clonality	Dilution	Source
Anti-tau DC25	Mouse monoclonal	1:1*	Axon Neuroscience (Bratislava, Slovak Republic)
Polyclonal anti-tau antibody	Rabbit polyclonal	1:5000	Axon Neuroscience (Bratislava, Slovak Republic)
Anti-β-tubulin (DC126)	Mouse monoclonal	1:1*	Axon Neuroscience (Bratislava, Slovak Republic)
Anti-tyrosinated tubulin	Rat monoclonal	1:1*	Axon Neuroscience (Bratislava, Slovak Republic)
Anti-DC39C	Mouse monoclonal	1:1*	Axon Neuroscience (Bratislava, Slovak Republic)
Anti-synaptophysin	Mouse monoclonal	1:3000	Synaptic Systems (Gottingen, Germany)
Anti-α-tubulin	Mouse monoclonal	1:2500	Synaptic Systems (Gottingen, Germany)
Anti-neuroligin	Mouse monoclonal	1:2000	Synaptic Systems (Gottingen, Germany)
Anti-drebrin	Rabbit polyclonal	1:2000	Abcam (Cambridge,UK)
Anti-acetylated–tubulin (6–11B–1)	Mouse monoclonal	1:3000	Abcam (Cambridge,UK)
Anti-bassoon (SAP7F407)	Mouse monoclonal	1:1000	Abcam (Cambridge,UK)
Anti-actin (ACTN05)	Mouse monoclonal	1:2500	Abcam (Cambridge,UK)
Anti-neurofilament antibody (SMI 312)	Mouse monoclonal	1:1000	Abcam (Cambridge,UK)
Amyloid precursor protein (22C11)	Mouse monoclonal	1:1000	Millipore (California, USA)
Anti-tubulin detyrosinated	Rabbit polyclonal	1:2000	Millipore (California, USA)
Anti-PSD95 (7E3–1B8)	Mouse monoclonal	1:2500	Thermo–scientific (Illinois USA)
Anti-GAP43	Mouse monoclonal	1:2500	Novus biological (Cambridge,UK)
Anti-MAP 2 (2a+2b)	Mouse monoclonal	1:2500	Sigma (Vienna,Austria)
Anti-tau pT205	Rabbit polyclonal	1:1000	Invitrogen (California, USA)
Anti-tau pT212	Rabbit polyclonal	1:1000	Invitrogen (California, USA)
Anti-tau pS214	Rabbit polyclonal	1:1000	Invitrogen (California, USA)
Anti-tau pS262	Rabbit polyclonal	1:1000	Invitrogen (California, USA)
Anti-tau pS356	Rabbit polyclonal	1:500	Abcam (Cambridge,UK)
Anti-tau DC11	Mouse monoclonal	1:100	Axon Neuroscience (Bratislava, Slovak Republic)
Anti-actin	Mouse monoclonal	1:2500	Abcam (Cambridge,UK)

* Supernatant from cultured hybridoma cells was used.

### Synaptosomal fractionation

Synaptosomal fractionation was performed according to previously published protocols (Hahn et al., 2009; Ciani et al., 2011) with minor modifications. Briefly, tissues were homogenized in Buffer A containing 0.32 M sucrose, 4 mM HEPES at pH 7.4 and protease inhibitor cocktail. Cell debris and nuclei were removed by centrifugation at 800 × g for 10 min and the resulting supernatant was collected (labeled S1). A fraction of S1 was stored and used as total protein extract. The pellet was re-suspended in buffer A and centrifuged at 800 × g for 10 min, the resulting supernatant and S1 were pooled and spun at 9000 × g for 15 min producing supernatant (S2) and pellet (P2). The pellet P2 was further suspended in buffer A and layered over a discontinuous sucrose gradient (0.8/1.0/1.2 M sucrose in 4 mM HEPES, pH 7.4) and spun at 65,000 × g for 45 min in MLS 50 rotor, Beckmann ultracentrifuge (Beckmann instrument Inc, California, US). The synaptosomal fraction at interface 1.0 M and 1.2 M sucrose was carefully collected and suspended in buffer B containing 0.32 M sucrose, 4 mM HEPES and 150 mM NaCl with phosphatase and protease inhibitors. The synaptosomal fractions were then incubated on ice in an equal volume of buffer C containing 1% Triton X-100, 0.32 M sucrose and 12 mM Tris at pH 8.0 for 15 min. After 15 min, the samples were centrifuged at 82,500 × g for 45 min. The triton extractable fractions (supernatant) represent the presynaptic fraction (synaptic membrane fraction (SMF)) containing the presynaptic proteins and the pellet correspond to postsynaptic density (PSD). All steps were performed in 4°C and aliquots of samples were stored in –80°C. Protein concentration was determined using BCA assay (Bio-rad laboratories Inc, California, USA) and equal amounts of proteins were loaded onto sodium dodecyl sulfate (SDS)-polyacrylamide gel electrophoresis (PAGE) gels. Anti-synaptophysin and anti-PSD95 antibodies were used to assess the purity of the fractions.

### Sarkosyl fractionation

Sarkosyl insoluble tau was extracted according to published protocols (Greenberg and Davies, 1990; Zilka et al., 2006). Right cortex of wild-type (wt) and transgenic animals were homogenized using OMNI homogenizer in buffer containing 20 mM Tris pH 7.4, 800 mM NaCl, 1 mM EGTA, 1 mM EDTA, 10% sucrose and protease inhibitors. After centrifugation at 20,000 × g for 20 min the supernatant (S1) was collected and small fraction was saved as total protein extract. Sarkosyl (40% w/v in water) was added to the final concentration of 1% and mixed by stirring for 1 h at room temperature. The samples were then centrifuged at 100,000 × g for 1 h at 25°C using Beckmann TLA 100 (Beckmann instrument Inc, California, USA). Pellets were washed in PBS and finally dissolved in SDS loading buffer to 1/50 volume of S1 and 20 μg w/v corresponding to S1 fraction was used for western blot analysis.

### Western blotting

Proteins were resolved on 12% SDS-PAGE and transferred to nitrocellulose membrane. The membranes were incubated in 5% non-fat free milk or BSA in 1 × TBS-Tween for 1 h. The blots were incubated with primary antibodies for 2 h at room temperature or overnight at 4°C. Blots were developed using enhanced chemiluminiscence western blotting detection system SuperSignal West Pico chemiluminescenscent Substrate (Thermo Scientific, USA) on Image Reader LAS-3000 (FUJI Photo Film Co, Ltd, Tokyo, Japan). Modified tubulin blots were stripped and re-probed using total α-tubulin antibody for quantitation. Actin was used as loading control and all relevant data were normalized to actin. The western blots (WB) were quantified using Advanced Image Data Analyzer software (AIDA Biopackage Raytest, Germany).

### Quantification of amyloid β using Elisa

Total synaptosomes were extracted as described earlier and used to quantify amyloid-β40 (Aβ40) and β42. Protein concentration was determined using BCA protein estimation and 400 μg of total synaptosomes were used for assay. Eight samples per group were analyzed. Amyloid β assay kits (WAKO, Japan) were used for assay and experiments in duplicates were performed according to manufacturer’s instructions.

### Confocal microscopy

Rats were perfused with phosphate saline buffer (PBS, pH 7.2) followed by 4% paraformaldehyde (PFA, pH 7.2), and the brains were removed. Tissues were postfixed in 4% PFA overnight, followed by treatment with 25% sucrose for 48 h to provide cryoprotection. Brains were then frozen in isopentane at −40°C. Coronal sections (40 μm) were cut in a cryostat at −18°C. Free-floating sections were incubated with primary antibodies (rabbit polyclonal anti-P-tau T205, mouse monoclonal DC11) overnight at 4°C. Sections were subsequently incubated with secondary antibodies conjugated with ALEXA 488 or ALEXA 546 fluorescent dyes (Invitrogen-Molecular Probes, Eugene, OR, USA) for 1 h at room temperature. After washing, the sections were mounted onto slides using Vectashield mounting medium (Vector Laboratories), and examined with an Olympus IX 71 Fluorview laser scanning confocal microscope.

### Electron microscopy

Rats (*n* = 3) were perfused with 300 mmol/l glutaraldehyde in 100 mmol/l cacodylate buffer, their brains were extracted and fixed in the same buffer overnight. Brains were cut to pieces (1 mm^3^), and postfixed in 40 mmol/l osmium tetroxide in 100 mmol/l cacodylate buffer (1 h). After rinsing in cacodylate buffer and dehydration in ethanol, samples were embedded in araldite resin (Durcupan ACM, Fluka). Ultrathin sections (60 nm thick) were cut using Leica EM UC6 ultramicrotome and stained with uranylacetate and lead citrate. Sections were examined under FEI Morgagni 268D electron microscope (FEI Company, Prague, Czech Republic) at 70 kV. Comparative images were captured using the same resolution as indicated in the EM micrographs.

### Data analysis

All experiments were repeated three times for consistency. Only one randomly selected value per experiment, however, was used for statistical analysis. Representative blots are shown. Statistical analyses were performed with R software (R Development Core Team, 2014). To avoid the pure asymptotic behavior of commonly used statistical methods, such as Student’s *t*-test or ANOVA, in our experimental design, we evaluated the differences between the means using a nonparametric bootstrap-*t* method (1000 bootstrap replications of the data) on the significance level *α* = 0.05. Bootstrap two-sample *t*-statistics were used as the test criterion. Additionally, we calculated the 95% bootstrap-*t* empirical confidence intervals (CI; Efron and Tibshirani, 1993) for the differences between the means. Statistical results are presented as bootstrap *p*-values, Monte-Carlo (MC) estimates of the mean differences, and MC estimates of upper and lower bounds of 95% CI. The statistical results are expressed by *p*-values, mean differences, and CI in the form “(lower bound, upper bound)”. If the *p*-value is greater or equal than 0.05, the CI includes zero; if the *p*-value is smaller than 0.05, the CI does not include zero. Graphs representing data points and mean for individual protein per group were generated using PRISM (GraphPad Software, Inc. California, USA), where *p ∈ 〈0.01, 0.05); **p ∈ 〈0.001, 0.01); ***p ∈ (0, 0.001) are used to indicate statistical significance.

## Results

### Cortical tau neurofibrillary pathology is a hallmark of the transgenic rat model of human tauopathy

**Figure 1:**
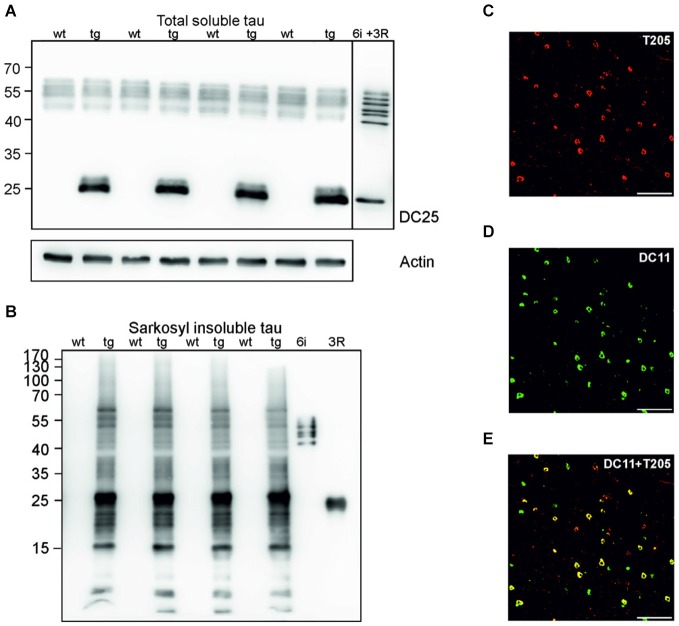
**Neurofibrillary degeneration in the cortex of transgenic rat model for human tauopathy. (A)** Western blots (WB), using pan-tau antibody DC25, showed no change in the expression of total endogenous rat tau in the cortex of transgenic rats (Tg) when compared to wild-type rats. Expression of transgenic human truncated tau (151–391) was similar in all tested transgenic animals. Actin was used as loading control. **(B)** Antibody DC25 recognizes extensive sarkosyl insoluble tau in the cortex of transgenic rats. No sarkosyl insoluble tau was observed in control animals. Recombinant tau isoforms (6i) and human truncated tau 3R (151–391) were used as controls. **(C)** Confocal microscopy revealed many neurofibrillary tangles containing phosphorylated tau at T205. **(D)** Many neurofibrillary tangles contained DC11 positive truncated tau. **(E)** Colocalization of pT205 and DC11 positive tangles (merged **C** and **D**). Scale bar = 100 μm.

Sarkosyl insoluble tau is considered the proteomic correlate of the mature neurofibrillary degeneration. We isolated both soluble and sarkosyl insoluble tau from the cortices of wild-type and transgenic animals. Using pan tau antibody DC25, we observed an identical pattern of endogenous rat tau in soluble fractions, and an additional presence of human truncated tau in the transgenic animals (Figure 1A). In the sarkosyl insoluble tau fractions isolated from transgenic rats, tau assembly comprised of higher and low molecular weight tau species. In the wild-type rat brain extracts sarkosyl insoluble tau was absent (Figure 1B). Transgenic rats developed extensive neurofibrillary degeneration in the cortex. Neurofibrillary pathology was composed of both phosphorylated (Figure 1C) and truncated tau species (Figure 1D). Confocal micrographs revealed that the majority of neurons contained tau phosphorylated at threonine 205 and DC11 positive truncated tau (Figure 1E).

### Truncated tau impaired synaptic tau proteome in the rat model of tauopathy

**Figure 2:**
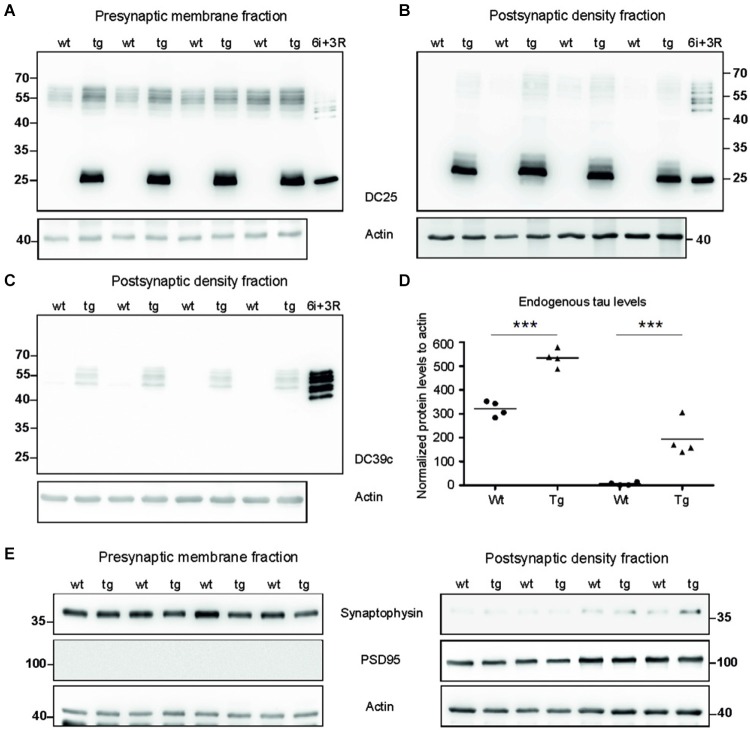
**Tau proteome was elevated and mislocalized in synapses of transgenic rats. (A)** WB analysis of presynaptic fractions using DC25 antibody revealed the presence of endogenous tau and truncated tau in the presynaptic compartments of wild-type and transgenic animals. Endogenous tau levels were, however, elevated in the presynaptic compartments of transgenic rats compared to wild-type rats. **(B)** WB analysis of PSD using DC25 antibody in wild-type and transgenic rats showed the presence of truncated tau in the PSD of transgenic rats. Very faint, if any, endogenous tau was observed in PSD of wild-type animals. Recombinant tau isoforms (6i) and truncated tau (3R) were used as controls in both **(A)** and **(B)**. **(C)** WB using DC39C—an antibody recognizing exclusively full length tau isoforms—confirmed the presence of endogenous rat tau in the post synaptic density of the transgenic rat cortex. Faint staining of tau was present in the PSD of wild-type rats. Recombinant tau isoforms (6i) were used as control. **(D)** Endogenous tau increased significantly in presynaptic as well as postsynaptic fraction. Graphs show individual data points and means for tau protein levels for each group in individual compartments. Statistical evaluation revealed changes in endogenous tau protein levels in the pre and postsynaptic fractions. Data represents three individual experiments (***p ∈ (0, 0.001)). **(E)** Isolated presynaptic and postsynaptic fractions were immunostained using anti-synaptophysin and anti-PSD95 antibodies for assessment of the purity of synaptic fractions. Pure synaptic fractions were observed in both wild-type and transgenic animals. Actin was used as loading control.

To investigate synaptic damage induced and driven by truncated tau we assessed the distribution of rat endogenous tau and human truncated tau in the synaptic membrane fraction (SMF) and postsynaptic density fraction (PSD). In SMF, pan tau antibody DC25 recognized endogenous rat tau in both wild-type and transgenic animals (Figure 2A). Postsynaptically, however, endogenous tau was present almost exclusively in PSD of transgenic rats, and only traces were observed in the PSD of wild-type animals (Figure 2B). We further confirmed the presence of endogenous tau in the postsynaptic fraction in transgenic rats using DC39C antibody that specifically recognizes the C-terminus of tau protein and thus stains only endogenous rat tau (Figure 2C). Human truncated tau was distributed in both synaptic fractions in transgenic animals (Figures 2A,B). Quantifying the levels of endogenous tau revealed a significant increase in rat tau isoforms in the pre-synaptic (*p* = 0.003; 215.38; (158.20, 272.56), Figure 2D) and post-synaptic compartments (*p* = 0.002; 150.79; (125.06, 176.52), Figure 2D) in transgenic rats.

We evaluated the purity of synaptic fractions using either synaptophysin—a specific marker for presynaptic compartment, or PSD95—a specific marker for the PSD (Figure 2E). Blots were probed with synaptophysin and then re-probed with PSD95. There was very little or no cross-contamination between the synaptic fractions.

### Synaptic truncated tau exhibited different phosphorylation patterns in pre- and postsynaptic compartments of transgenic animals

**Figure 3:**
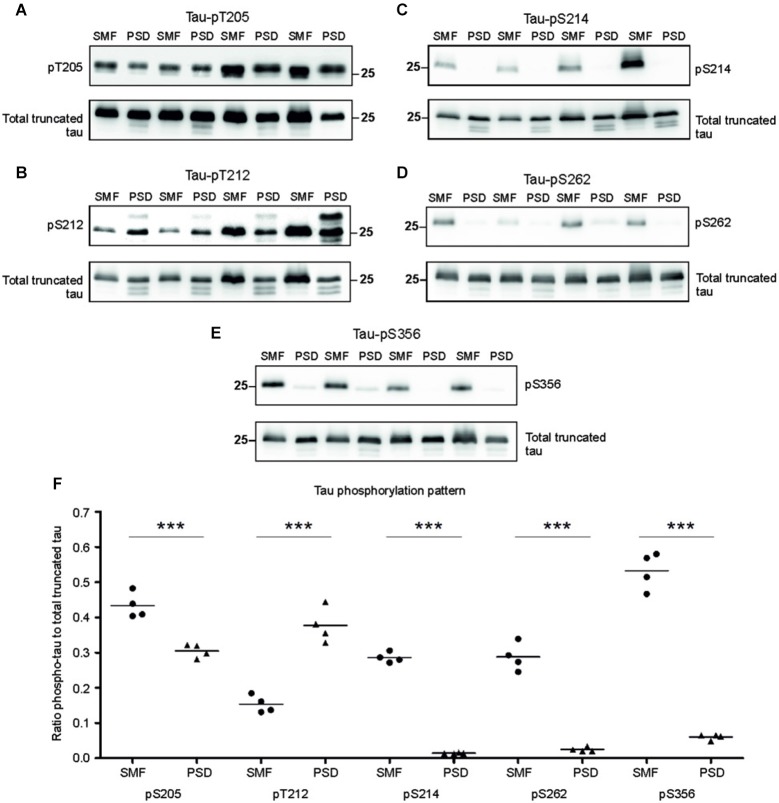
**Phosphorylation pattern of tau proteome was different in presynaptic and postsynaptic compartments of transgenic animals**. Immunoblots of presynaptic and postsynaptic fractions from transgenic animals using antibodies against phosphorylated tau at **(A)** threonine 205 (pT205), **(B)** threonine 212 (pT212), **(C)** serine 214 (S214), **(D)** serine 262 (S262), **(E)** serine 356 (S356). For relative quantification, each blot was stripped and reprobed with polyclonal anti-tau (total tau). **(F)** Blots revealed increased phosphorylation of tau at residues T205, S214, S262, and S356 in the presynaptic compartments, and increased phosphorylation at T212 in the postsynaptic fraction. Graph showing data points and means for specific tau-phospho epitopes in individual groups. Statistical evaluation revealed differences in phospho-tau levels from WB (***p ∈ (0, 0.001)).

Phosphorylation regulates microtubule binding activity of the tau protein. In AD brains, site specific phosphorylation of tau correlates with neuronal pathology (Augustinack et al., 2002). Therefore, we evaluated tau phospho-pattern in the synaptic compartments of transgenic animals. We focused on phospho tau epitopes that play important role in AD namely—T205, T212, S214, S262, and S356. We selected antibodies specifically recognizing these tau phospho-sites and investigated the phospho-status of truncated tau in the presynaptic fraction (SMF) and PSD fraction of transgenic rats (Figures 3A–D). The blots were stained with respective phospho-antibodies, stripped and re-stained using polyclonal anti-tau antibody (poly-tau). Phospho-tau levels were normalized to total tau levels.

Densitometric analysis revealed significant differences in tau phospho-pattern in the synaptic fractions (Figure 3E). Interestingly, antibodies recognizing phosphorylation at T205, S214, S262 and S356 displayed higher immunoreactivity in the presynaptic fraction (pT205, *p* = 0.006; –0.16; (–0.218, –0.095); pS214, *p* = 0.003; –0.28; (–0.304, –0.258); pS262, *p* = 0.009; –0.27; (–0.325, –0.221); pS356, *p* = 0.006; –0.39; (–0.545, –0.239)), while antibody specific for phospho-T212 showed increased immunoreactivity in the PSD when compared to SMF (*p* = 0.008; 0.23; (0.163, 0.305)) (Figure 3F).

### Truncated tau selectively damaged cytoskeletal proteins in pre- and postsynaptic compartments of transgenic rats

**Figure 4:**
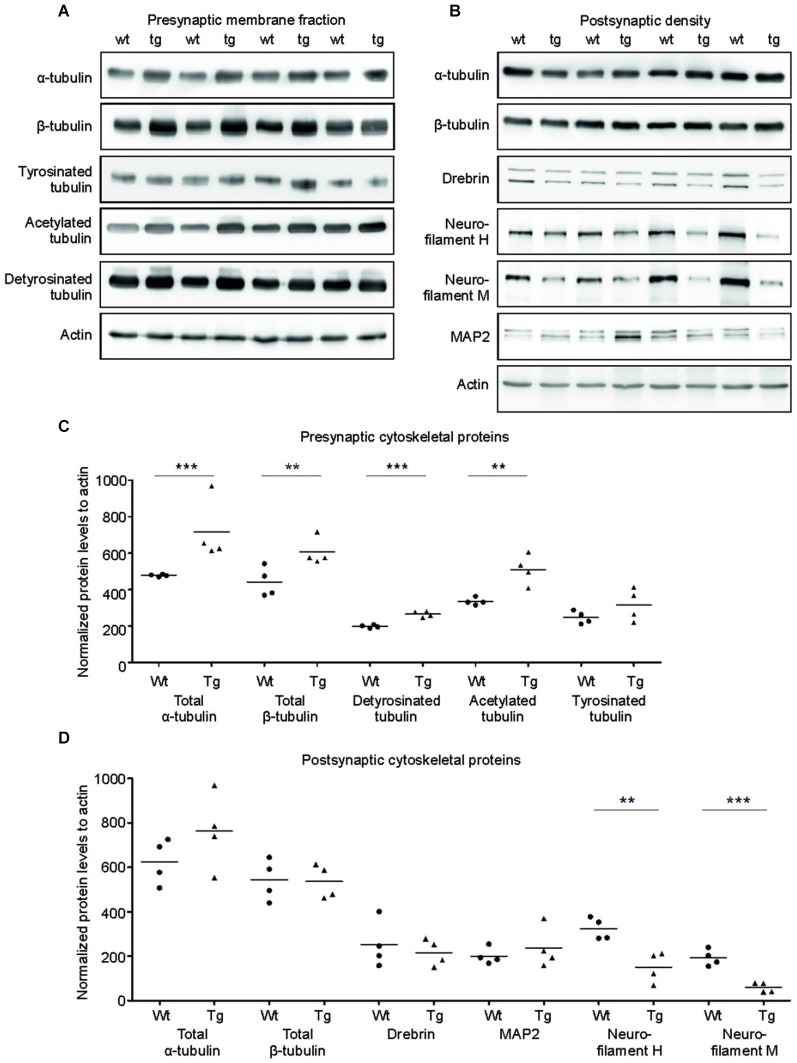
**Effect of truncated tau on cytoskeletal proteins in presynaptic and postsynaptic compartments of transgenic rats. (A)** The levels of acetylated and detyrosinated tubulin increased selectively in the presynaptic fraction. WB of presynaptic fractions from wild-type (wt) and transgenic (tg) rats with specific tubulin antibodies as indicated. Modified tubulin levels were normalized to total tubulin levels. Actin was used as loading control. **(B)** Presynaptic levels of α-tubulin and β-tubulin along with acetylated-tubulin and detyrosinated tubulin increased, whereas levels of tyrosinated tubulin did not change. **(C)** Levels of neurofilament proteins decreased in PSD of transgenic rats. WB comparing cytoskeletal proteins of postsynaptic densities of wild-type (wt) and transgenic (tg) rats. Actin was used as loading control. **(D)** Postsynaptic levels of total tubulin protein levels (α/β) did not change, whereas levels of neurofilaments H and M decreased in transgenic rats when compared to wild-type rats. Significant decrease in levels of neurofilament H and neurofilament M in the postsynaptic densities of transgenic rat compared to wild-type. In both **(B)** and **D)**, graphs show data points and the mean values for individual pre- or postsynaptic cytoskeletal proteins per group (**p ∈ 〈0.001, 0.01); ***p ∈ (0, 0.001)).

Tau is a major microtubule binding protein localized mainly in the axons, and plays an important role in microtubule polymerization and stability. Therefore we investigated the pathological effect of truncated tau presence on cytoskeletal proteins in the presynaptic compartments. Strikingly, we observed a significant increase in levels of total α-tubulin (*p* = 0.002; 154.97; (100.71, 209.23)) and a sizeable increase in levels of total β-tubulin (*p* = 0.048; 139.15; (14.17, 264.12)) in the SMF fractions of transgenic animals when compared with wild-type rats (Figures 4A,C).

We next investigated whether the specific increase of tubulin might affect the dynamic instability of microtubules in the presynaptic compartment in transgenic rats. Microtubule polymerization is associated with several posttranslational modifications of tubulin and specific modifications influence the microtubule stability *in vivo* (Verhey and Gaertig, 2007). To investigate posttranslational modifications of synaptic tubulin, we used antibodies specific to tyrosinated tubulin—a marker for unstable microtubules (Paturle et al., 1989; Marcos et al., 2009), and detyrosinated and acetylated tubulin—markers for stable microtubules (Webster et al., 1987; Hammond et al., 2008; Figures 4A,C). We found a significant increase in detyrosinated (*p* = 0.006; 65.06; (40.76, 89.37)) and acetylated tubulin (*p* = 0.015; 166.63; (64.77, 268.48)) in the presynaptic compartments of the transgenic rats compared to wild-type rats. However, we observed no significant change in the levels of tyrosinated tubulin when compared to wild-type rats (*p* = 0.209; 70.49; (–103.20, 244.18)).

In the PSD, truncated tau-induced cytoskeletal changes were different than in the SMF. We detected no significant changes in the levels of total α-tubulin or total β-tubulin in the PSD (Figures 4B,D; total α-tubulin *p* = 0.224; 144.46; (–85.59, 374.52); total β-tubulin *p* = 0.902; –12.71; (–145.13, 119.70)). In addition, we did not observe any change in acetylated tubulin levels in the PSD (data not shown). Furthermore, the levels of MAP2—a dendrite specific microtubule binding protein also remained unaltered. However, we found significant changes in neurofilament proteins (Figures 4B,D). Neurofilaments represent a major component of the PSD, and are localized in close proximity to the PSD membrane (Blomberg et al., 1977; Liu et al., 2006). We observed a significant decrease in neurofilament H (*p* = 0.017; –181.91; (–281.23, –82.59)) and neurofilament M (*p* = 0.006; –141.06; (–196.73, –85.39)). Additionally, we also observed a slight nonsignificant decrease in drebrin E/A (*p* = 0.578; 79.85; (–73.75, 233.45)), a dendrite-specific actin-binding protein, that regulates spine morphology and size levels (Ivanov et al., 2009).

### Truncated tau deregulated synaptic markers only in presynaptic compartments in transgenic rats

**Figure 5:**
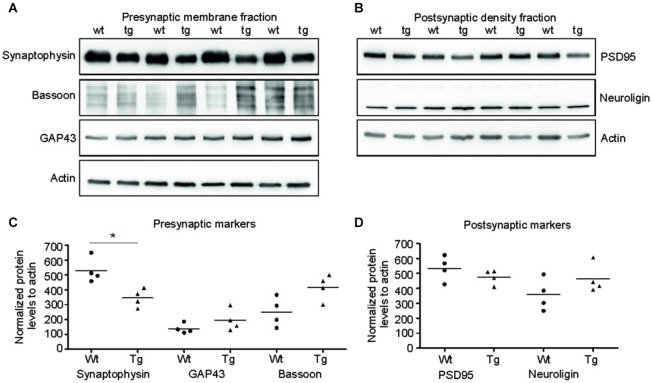
**Truncated tau deregulated selected synaptic proteins in rat model. (A)** Immunoblots of presynaptic fractions with antibodies against synaptophysin, Bassoon and GAP 43. Actin was used as loading control. **(B)** Graphs show data points and the mean values for the presynaptic proteins per group. Statistical evaluation of presynaptic proteins revealed decrease in synaptophysin levels (*p < 0.05), no change in bassoon levels, and no change in GAP 43 protein levels. **(C)** Western blots of postsynaptic fraction with PSD95 and neuroligin indicated no change in the levels of these proteins when compared to wild-type rats. **(D)** Graphs show data points and mean values of postsynaptic proteins for individual groups. Statistical evaluation of postsynaptic proteins revealed no significant change in PSD 95 and neuroligin levels.

Several presynaptic and postsynaptic proteins are deregulated in human AD and other tauopathies. To study the effect of truncated tau presence on synaptic markers, we assessed the levels of selected presynaptic and postsynaptic proteins in synaptic fractions (Figures 5A,C). Western blotting analysis revealed significant decrease in synaptophysin levels in the presynaptic fraction of transgenic rats in comparison to wild-type animals (*p* = 0.026; –211.81; (–328.96, –94.65)). In contrast to synaptophysin, we observed only nonsignificant increase in bassoon—a synaptic vesicle clustering protein (*p* = 0.056; 137.73; (−25.38, 300.86)), and no change in the levels of GAP 43 protein, a neurite growth cone protein (*p* = 0.278; 81.67; (−24.36, 187.71)). In the PSD, we did not detect any significant changes in the levels of PSD95 and neuroligin, a ligand for presynaptic β-neurexins (Figures 5B,D; PSD95: *p* = 0.284; –51.80; (–161.13, 57.53), Neuroligin: *p* = 0.197; 111.42; (–43.03, 265.88)) (Figures 8A,B).

**Figure 6:**
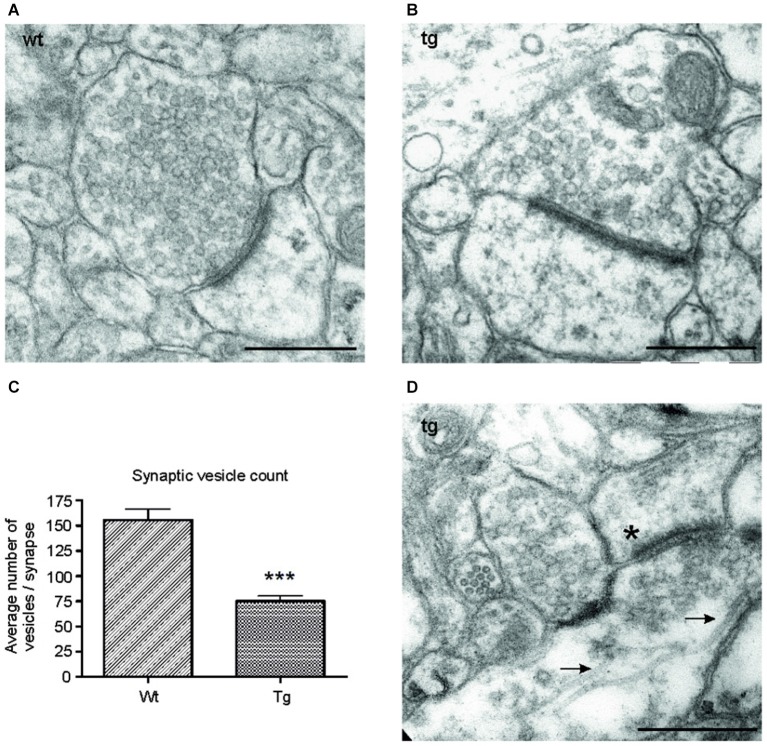
**Number of synaptic vesicles was reduced in synapses of transgenic animals**. Representative electron micrographs of synapses from **(A)** wild-type (wt), and **(B)** transgenic (tg) rats. **(A)** Synapses in wild-type rats showed an intact synaptic junction and presence of numerous synaptic vesicles. **(B)** Electron micrographs of synapses from transgenic rats revealed a decrease in the number of synaptic vesicles. **(C)** The number of synaptic vesicles decreased significantly in transgenic animals. Graph shows the difference between synaptic vesicle count/synapse in wild-type and transgenic animals (***p ∈ 〈0.001, 0.01)). **(D)** Electron micrographs from transgenic animals revealed the presence of microtubule bundles (arrows) in presynaptic terminal of transgenic animals. The asterisk indicates the postsynaptic terminal. Scale bars for micrographs = 500 nm.

**Figure 7:**
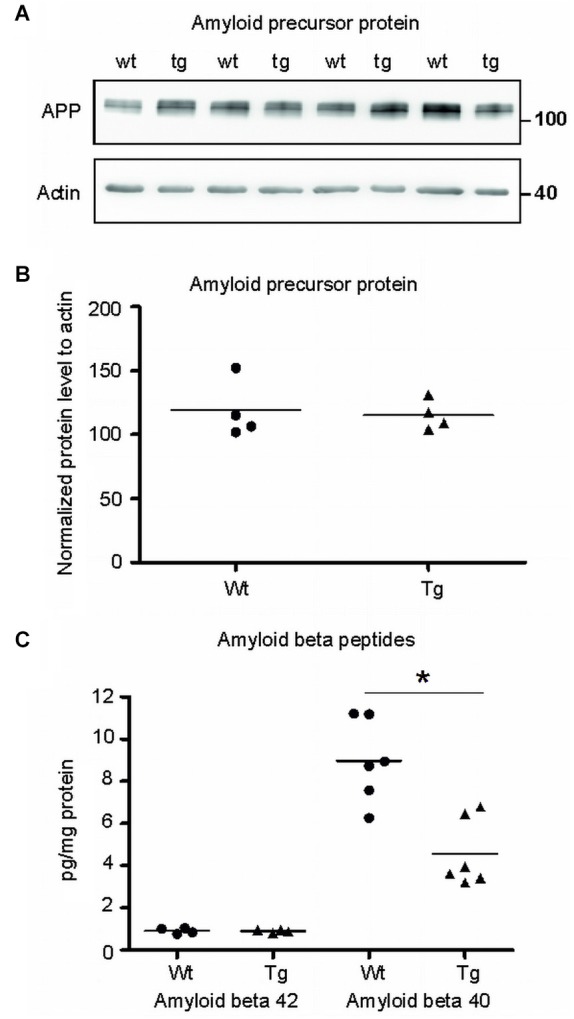
**Amyloid-β 40 was reduced in the synapses of transgenic animals but not amyloid-β 42. (A)** Immunoblot using N- terminal antibody of APP showed no change in the levels of APP in transgenic rats (tg) with respect to wild-type (wt) rats. Actin was used as loading control. **(B)** Levels of amyloid precursor protein did not change. **(C)** Graphs show data points and means of individual groups for the Aβ42 and Aβ40 peptides. Levels of amyloid-β alloforms in total synaptosomes showed no change in Aβ42 and a significant decrease in Aβ40 (*p ∈ 〈0.01, 0.05)).

**Figure 8:**
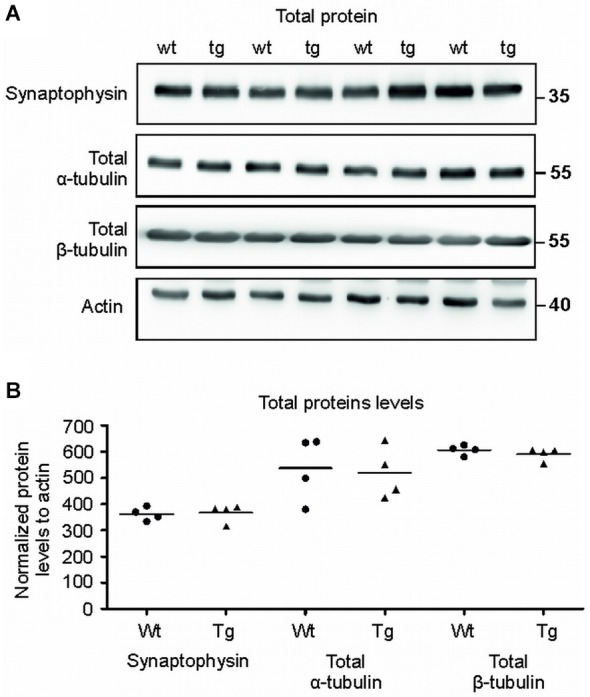
**Total protein levels of synaptophysin and tubulins were unaltered. (A)** Immuno blots of total protein extracts from wild-type (wt) and transgenic animals (tg) against total synaptophysin, total α-tubulin, total β-tubulin proteins. Actin was used as loading control. **(B)** Graph shows data points and mean for tubulin proteins and synaptophysin in each group. No significant changes in the protein levels were observed.

### Truncated tau reduced number of synaptic vesicles and induced formation of microtubular bundles

To investigate the ultrastructural changes in the synapses of transgenic animals *in vivo*, we examined cortical sections of wild-type and transgenic animals using electron microscopy (EM). EM micrographs revealed a decrease in synaptic vesicles in the synapses of transgenic animals (Figures 6A,B). To further characterize the changes in synaptic vesicles, we counted the number of synaptic vesicles per synapse in randomly selected sections in the wild-type and transgenic animals. Synaptic vesicles from 60 synapses/animal exhibiting preserved junctions were counted in wild-type (*n* = 3) and transgenic animals (*n* = 3) by a blind observer. The number of vesicles per synapse decreased significantly in transgenic rats compared to wild type rats (Figure 6C; *p* < 0.0001; –44.05; (–53.27, –34.82)), corresponding to the decrease in synaptophysin levels (Figure 5C). Consistent with the results obtained from synaptic fractionation, we observed microtubule bundles in the presynaptic terminals of transgenic animals (Figure 6D).

### Truncated tau-mediated deregulation is not dependent on Aβ

To identify the position of amyloid cascade in truncated tau-mediated synaptic deficits, we compared the levels of APP, amyloid-β 42 (Aβ42) and Aβ40 in synapses of transgenic and wild-type animals. Staining using anti APP antibody (clone 22C11) revealed no change in the levels of total APP in the transgenic animals (Figures 7A,B; *p* = 0.868; –84.30; (–419.99, 251.39)). Furthermore, we quantified the levels of amyloid peptides Aβ40 and Aβ42 in total synaptosomes by quantitative ELISA. The quantification did not reveal any changes in the levels of toxic Aβ42 in transgenic animals when compared to wild-type rats (Figure 7C; *p* = 0.878; 0.01; (–0.22, 0.24)). The levels of Aβ40 were, however, significantly reduced (*p* = 0.022; –2.86 ; (–5.07, –0.64)) in synapses of transgenic animals.

### The levels of synaptophysin and tubulin in total brain extracts showed no changes in transgenic animals when compared to controls

To verify that the observed differences were specific for synapses, we also quantified the levels of selected proteins in total brain extracts (Figures 8A,B). Immuno-quantification of total protein extract revealed no changes in the levels of synaptophysin (*p* = 0.633; –3.31; (–48.64, 42.01)), α- tubulin (*p* = 0.822; 24.82; (–175.76, 225.41)) and β-tubulin (*p* = 0.376; –22.22; (–55.21, 10.76)) suggesting that the pathological changes were present exclusively in the synaptic terminals of the transgenic animals.

## Discussion

Synaptic failure and neurofibrillary tangles strongly correlate with cognitive decline in AD (Terry et al., 1991; Samuel et al., 1994). Growing body of research has demonstrated that AD brains show a marked reduction in synaptic density and a loss of dendritic spines in cortex and hippocampus (DeKosky and Scheff, 1990). The loss of synaptic proteins and the reduction of dendritic processes in AD were also attributed to tau neurofibrillary pathology (Callahan and Coleman, 1995; Falke et al., 2003; Ramsden et al., 2005; Kambe et al., 2011). Several recent reports have demonstrated that microtubule associated protein tau induced synaptic impairment. This process is mainly regulated by N-methyl-D-aspartate receptor-dependent tau phosphorylation (Ittner et al., 2010; Mondragón-Rodríguez et al., 2012). However, these studies focused mainly on the post-synaptic compartment and the toxic effects of misfolded tau on the pre-synaptic compartment are poorly understood. Furthermore, data on the pathological tau proteome in the synaptic compartments are missing.

To unravel the pathological signature of truncated tau in both synaptic compartments we utilized rat model for human tauopathy, expressing truncated tau in the central nervous system. The rats express express six tau isoforms in the brain, similar to humans (Hanes et al., 2009), and represent an excellent model for human AD. Moreover, the rat model develops an extensive neurofibrillary degeneration in the cortex that fulfills the criteria for human AD neurodegeneration, including argyrophilia, thioflavin S positivity and Congo red birefringence (Filipcik et al., 2012). The synaptic biology, signaling and proteome have also been extensively studied in these rats, which enabled us to study the pathological changes of synaptic structure in a well characterized animal model. Furthermore, we used a well-established synaptic fractionation protocol that has been applied extensively to investigate the biology and proteome of synaptic fractions (Cohen et al., 1977; Rebola et al., 2003; Zhou et al., 2007; Ciani et al., 2011; Rodrigues et al., 2014; Sahara et al., 2014) and to examine post-mortem human brains from AD (Tai et al., 2012) and other neuropsychiatric diseases (Hahn et al., 2009).

Here we demonstrated that rat tau proteins were distributed differentially in synaptic compartments in transgenic and age-matched control rats. Specifically, in control rats, the amount of the endogenous rat tau in post-synaptic compartment was significantly lower than in pre-synaptic compartment. These results are consistent with a recent study showing synaptic localization of tau in human brains (Tai et al., 2012) and corroborate the similarity between rat and human synaptic tau proteome. In transgenic rat brains, we observed a significant increase in tau protein in the post-synaptic density when compared to age-matched controls. Similarly, increase in tau protein along with tau aggregation was detected in the PSD of AD brain (Fein et al., 2008; Tai et al., 2012). This suggests that the normal sorting mechanism of tau protein is compromised in the transgenic animals, similar to AD brains. These results also show that tau mis-sorting may represent a key step in the tau mediated neurodegeneration.

Synaptic tau proteome in transgenic animals exhibited different phosphorylation patterns in the pre- and postsynaptic compartments. In transgenic animals, truncated tau in the presynaptic compartment was heavily phosphorylated in the microtubule binding domain and the proline rich region in comparison to the PSD. An identical pattern has been suggested by several studies indicating that pathological phosphorylation of tau begins in the axons (Papasozomenos and Su, 1991; Khatoon et al., 1994; Andorfer et al., 2003). Our results further show that tau hyperphosphorylation displays distinct pattern in the pre- and postsynaptic compartments, which suggest that different phospho-tau species are distributed in SMF and PSD.

The difference in the composition of truncated tau proteome in synaptic compartments may be responsible for the pattern of damage in the pre- and postsynaptic compartments. In presynaptic terminals, truncated tau species impaired dynamic microtubular instability leading to formation of microtubule bundles. These results are consistent with our previous findings, in which we demonstrated the formation of abnormally thick microtubules *in vitro*, after incubation of tubulin with truncated tau (Zilka et al., 2006). Furthermore, the expression of tau protein resulted in increased tubulin acetylation (Takemura et al., 1992; Perez et al., 2009), which could be one of the factors leading to microtubule bundling in the presynaptic terminals. In addition, several unknown factors may contribute to tau-induced microtubule bundling *in vivo* (Lee and Rook, 1992). Interestingly, a fraction of truncated tau in the presynaptic compartment was phosphorylated at T205, S214, pS262, and S356. Since phosphorylation at these residues detaches tau from microtubules (Fischer et al., 2009) the specific enrichment of these phospho-tau residues in the presynaptic terminals may prevent tau from binding to microtubules, possibly indicating a compensatory mechanism to minimize microtubule bundling. Highly stable microtubules, however, may impair the mobility of synaptic vesicles. Indeed, here we documented a significant decrease in the number of vesicles in the presynaptic terminals of transgenic rats when compared to controls. This reduction was also reflected by decrease in the levels of synaptophysin, a membrane protein of synaptic vesicles. Decrease in the number of vesicles in the synaptic terminals of transgenic animals may lead to decreased neurotransmitter release and in turn affect inter-neuronal synaptic dialog. However, the total synaptophysin and tubulin levels remained unchanged suggesting that the deregulation in these proteins occurs mainly in the presynaptic terminals.

In contrast to presynaptic terminals, truncated tau did not affect microtubule stability in the postsynaptic terminals. This difference may be attributed to the regional binding affinity of tau and the preference to axonal tubulin binding activity in neurons (Kanai and Hirokawa, 1995). Although microtubules were unaffected, the levels of cytoskeletal proteins, neurofilaments H and M, were significantly altered in the PSD. Decrease in neurofilaments has been reported in the early and late stages of AD (Kittur et al., 1994; Bajo et al., 2001; Counts et al., 2006). Neurofilaments regulate transport of cytoskeletal components, are tightly linked to the dendritic arborization in adult neurons, and play a crucial role in dendritic spine morphogenesis (Willard and Simon, 1983). Therefore, impaired structural assembly of neurofilaments may also alter excitatory and inhibitory synaptic activity.

Mocanu et al. (2008) showed that expression of truncated tau fragment K18 containing mutation ΔK280 in the pro-aggregation mice led to the reduction of dendritic spines and downregulation of synaptophysin in the hippocampal area CA1–CA3. The K18 tau fragment (Gln244-Glu372) is involved in the tau fragment used in this study (Ile151-Glu391). Both fragments contained structural determinants on mis-disordered tau protein that are essential for pathological tau–tau interaction (Kontsekova et al., 2014). However, it is important to note, that there is no tau mutation in AD and therefore our transgenic rat model expressing truncated tau with no tau mutation may better reflect tau pathological modification relevant for AD.

In order to delineate the role of amyloid-β in synaptic failure driven by truncated tau, we quantified the amounts of the most abundant amyloid beta alloforms Aβ42 and Aβ40 in synaptosomes. In human brain, the peptides co-exist under normal physiological conditions in an Aβ42:Aβ40 ratio of ~1:9 (Pauwels et al., 2012). We observed a similar ratio in our control rats. However, in transgenic rats this ratio was altered due to the decrease in Aβ40 levels and no detectable changes in the levels of Aβ42. It has been shown that Aβ40 is less neurotoxic than Aβ42 (Jan et al., 2008). The amyloidogenic activity of Aβ42 is 10-fold higher than that of Aβ40 (Matsuzaki, 2011) and their modes of oligomerization are distinct (Bitan et al., 2003). Furthermore, oligomeric Aβ40 did not induce inhibition of synaptic transmission unlike oligomeric Aβ42 (Moreno et al., 2009). Here we showed that the levels of Aβ42 were unaltered in the cortex of transgenic animals, demonstrating that the pathological changes associated with truncated tau are independent from Aβ42 toxicity. On the other hand, the truncated tau protein significantly altered the levels of Aβ40. Because Aβ40 is involved in long term potentiation (Wu et al., 1995), we hypothesize that truncated tau impedes synaptic activity via dysregulation of Aβ40 pathway. This suggests that amyloid β changes arise downstream of truncated tau pathology in the transgenic animals.

Detailed evaluation of functional consequences of truncated-tau on synaptic transmission, however, is beyond the scope of this study. Here we focused solely on the structural synaptic damage associated with the expression of truncated tau in the cortex of transgenic rats. Whether the observed changes in synaptic proteome have an immediate or gradual differential impact on synaptic transmission, or whether the expression of tau damages all or only a subset of synapses for a given neuron are important question which deserve a further study. However, recent study demonstrates that truncated tau (aa 1–369) is sufficient to induce synaptic and dendritic loss and reduce the field excitatory post-synaptic potential leading to the cognitive decline (Zhang et al., 2014).

Finally, we cannot completely exclude the effect of tau overexpression on the synaptic damage, however we can see specific pattern of synaptic damage in the cortex of transgenic rats. First of all, not all proteins are affected. Furthermore microtubular network is mainly damaged in the presynaptic compartment while neurofilaments are altered in postsynaptic compartment. It is important to mention that overexpression of truncated tau is similar in both compartments. Therefore we suggest that dysregulation of synaptic markers are caused by tau truncation rather than tau overexpression.

In conclusion, truncated tau protein displayed different phospho-patterns in the pre- and postsynaptic compartments, which was associated with selective damage of microtubular network in the presynaptic compartment and deregulation of neurofilaments in the post-synaptic density. Importantly, although the truncated tau protein orchestrated the synaptic deficits independently from Aβ42, it significantly regulated Aβ40. The study opens new avenues for better understanding of synaptic pathology in AD and related neurodegenerative diseases.

## Author contributions

Santosh Jadhav performed isolation of synaptic fractions, sarkosyl fractionation, western blotting experiments, and analysis of EM images. Norbert Zilka designed the protocol of the study. Santosh Jadhav, Norbert Zilka and Andrej Kovac performed EM experiments. Stanislav Katina did the statistical analysis. Santosh Jadhav and Zuzana Kazmerova performed ELISA experiments. Santosh Jadhav, Norbert Zilka and Michal Novak wrote the paper. All authors read and accepted the manuscript.

## Conflict of interest statement

The authors declare that the research was conducted in the absence of any commercial or financial relationships that could be construed as a potential conflict of interest.
